# Reducing Frequent Visits to the Emergency Department: A Systematic Review of Interventions

**DOI:** 10.1371/journal.pone.0123660

**Published:** 2015-04-13

**Authors:** Lesley J. J. Soril, Laura E. Leggett, Diane L. Lorenzetti, Tom W. Noseworthy, Fiona M. Clement

**Affiliations:** 1 The Department Community Health Sciences, Teaching Research and Wellness Building, 3280 Hospital Drive NW, Calgary, Alberta, Canada; 2 Institute for Public Health, Teaching Research and Wellness Building, 3280 Hospital Drive NW, Calgary, Alberta, Canada; 3 Institute of Health Economics, 1200, 10405 Jasper Avenue, Edmonton, Alberta, Canada; University of California San Diego, UNITED STATES

## Abstract

**Objective:**

The objective of this study was to establish the effectiveness of interventions to reduce frequent emergency department (ED) use among a general adult high ED-use population.

**Methods:**

Systematic review of the literature from 1950-January 2015. Studies were included if they: had a control group (controlled trials or comparative cohort studies), were set in an ED or acute care facility, and examined the impact of an intervention to reduce frequent ED use in a general adult population. Studies reporting non-original data or focused on a specific patient population were excluded. Study design, patient population, intervention, the frequency of ED visits, and costs of frequent ED use and/or interventions were extracted and narratively synthesized.

**Results:**

Among 17 included articles, three intervention categories were identified: case management (n = 12), individualized care plans (n = 3), and information sharing (n = 2). Ten studies examining case management reported reductions in mean (-0.66 to -37) or median (-0.1 to -20) number of ED visits after 12-months; one study reported an increase in mean ED visits (+2.79); and one reported no change. Of these, 6 studies also reported reduced hospital costs. Only 1 study evaluating individualized care plans examined ED utilization and found no change in median ED visits post-intervention. Costs following individualized care plans were also only evaluated in 1 study, which reported savings in hospital costs of $742/patient. Evidence was mixed regarding information sharing: 1 study reported no change in mean ED visits and did not examine costs; whereas the other reported a decrease in mean ED visits (-16.9) and ED cost savings of $15,513/patient.

**Conclusions:**

The impact of all three frequent-user interventions was modest. Case management had the most rigorous evidence base, yielded moderate cost savings, but with variable reductions in ED use. Future studies evaluating non-traditional interventions, tailoring to patient subgroups or socio-cultural contexts, are warranted.

## Introduction

Emergency departments (ED) are integral to providing rapid access to care for those with acute medical needs. However, frequent utilization of the ED is a challenging and contentious issue for clinicians and policy-makers.[1–3] Between 1 and 5% of the overall patient population seen in the ED account for approximately 12 to 18% of all annual ED visits.[4–9] This marginal proportion of the ED patient population, commonly referred to as ‘frequent ED users’, have complex healthcare needs that are not optimally managed within the context of the ED healthcare setting.[4–9] While some have speculated that the frequent ED visits by this patient population are preventable, and likely attributable to misuse of ED services for non-emergent care needs,[10,11] others have argued that upstream social and chronic health issues of frequent ED users are being mishandled or go unnoticed within the transactional care and episodic setting of the ED.[12]

Frequent users of the ED have also been described as frequent users of primary[8,9] and acute[8,9,11,13,14] health services. Such high health service use beyond the ED setting suggests that appropriateness of care, rather than access to care and/or attachment to an identified healthcare provider, may be a primary issue for these patients.[1,15] Many reviews of current approaches to managing frequent ED users commonly speak to the importance of the ED setting, claiming that few settings outside of the ED can provide a parallel level of, and rapid access to, urgent care.[16,17] The ED is, however, a costly setting for the delivery of chronic, continuing care. Accounts of high recurrent health resource use elicit concern over the potential excessive healthcare costs incurred by these patients.

A number of interventions aimed at reducing the number of ED visits by frequent users have been evaluated in the literature.[5,6] Previous systematic reviews of interventions for frequent ED users have described patient-centered programs predicated on interdisciplinary team approaches,[18–24] as those most commonly evaluated. Given the evolving nature of the frequent ED use and its persistent literature base, these previous reviews, unfortunately, offer perspectives that are either dated[5] or of limited scope (e.g. focused on a singular intervention-type and outcome).[6] Novel efforts to systematically evaluate the most current, broadest, and highest-quality evidence concerning all interventions for frequent ED use—examining the clinical, cost and contextual perspectives—are required.

The objective of this research was to establish the effectiveness of interventions aimed at reducing the ED utilization, in comparison to usual care, for individuals who are frequent users of the ED. This timely work is of particular importance to informing healthcare practitioners and decision-makers of the most clinical- and cost-effective intervention(s) for the development of appropriate policies to not only optimize the quality of care for, but also control very high healthcare costs incurred by, frequent ED users.

## Materials and Methods

A systematic review was completed to identify the published literature reporting the clinical efficacy or effectiveness and the related cost impact of interventions for a general, adult population of frequent ED users. This research was completed in accordance with the Preferred Reporting Items for Systematic Reviews and Meta-Analyses (PRIMSA; S1 PRISMA Checklist).[25] No protocol exists for this systematic review. As this research did not involve participation of human subjects, research ethics board review of the protocol for this systematic review was not required, nor was informed consent.

### Search strategy

The comprehensive search strategy was developed by an Information Specialist. EMBASE, MEDLINE, PubMED, the Cochrane CENTRAL Registry of Controlled Trials, and the Cochrane Database of Systematic Reviews were searched from 1950- January 26^th^, 2015. The EMBASE electronic search strategy is outlined in S1 Table. Terms such as “emergency service,” “emergency department,” “health care,” and “health service” were combined using the Boolean operator “and” with terms such as “frequent,” “over-use*,” “overutiliz*,” and “super-user.” Results were limited to English language, and excluded comments, editorials and letters. No other limitations were used.

### Selection of Literature

Studies were included if: they reported original data; had a control group (controlled trials or prospective comparative cohort studies); were set in an emergency department or acute care facility; focused on a general adult frequent ED user population; and, examined the impact of an intervention to reduce the ED utilization of frequent ED users. No fixed definition of frequent user was applied; any definition used within the included studies was accepted. Studies were excluded if they did not meet the criteria above, or if they only assessed a specific demographic or clinical group of frequent users (e.g. seniors, those with asthma, migraine suffers, homeless). References lists of previous systematic reviews were also hand-searched for relevant articles not identified in the search of the literature. All screening was completed in duplicate, with any disagreement between reviewers resolved through discussion and consensus, or through consultation with a third reviewer. A kappa statistic for agreement was calculated.

### Data Extraction and Analysis

Data from the included studies was extracted in duplicate using standardized data extraction forms. Any discrepancies in data extraction were resolved through consensus and discussion. For all studies, study type, publication date, country, number of participants, health care setting, definition of “frequent user”, type of intervention and comparator(s), and the primary outcome of interest (e.g. the reduction in ED visits between groups or pre- and post-intervention) were extracted. For studies that did not report the change in ED visits, the difference in median or mean number of ED visits was calculated where possible. The secondary outcomes of cost, related to the implementation of the intervention and/or hospital charges before and after the intervention, were also extracted when available. For studies that did not report change or difference in costs, the difference in per-patient and/or total hospitalization costs before and after the intervention were also calculated, where possible. Costs were primarily reported in United States Dollars (USD; $) or Euros (EUR; €) based on the study setting; any exceptions were otherwise noted. Heterogeneity in the study populations, healthcare settings, intervention and comparator types, as well as reported outcomes, prohibited meta-analysis of study outcomes. All data analyses (i.e. calculation of kappa statistic, change in ED visits and cost outcomes) were conducted using STATA 13.1 IC (College Station, Texas, USA).

### Quality Assessment

The Cochrane Risk of Bias Checklist was used to evaluate the quality of the included RCTs.[26] Broadly, seven areas of risk of bias are included in this checklist (random assignment generation; allocation concealment; blinding of participants and personnel; blinding of outcome assessment; incomplete outcome data; selective reporting; and any additional potential sources of bias) with each area given either “low,” “high,” or “unclear” risk of bias.[26] Comparative cohort studies were assessed using the Downs and Black Checklist.[27] This checklist includes 27 criteria, widely covering areas reporting quality, external and internal validity, and power[27]. Studies are assigned a value of “1” if they meet the question criteria, and “0” if they do not or if it is not possible to determine; with one exception where one question may be given “2” points.[27]

## Results

### Characteristics of Included Studies

**Fig 1:**
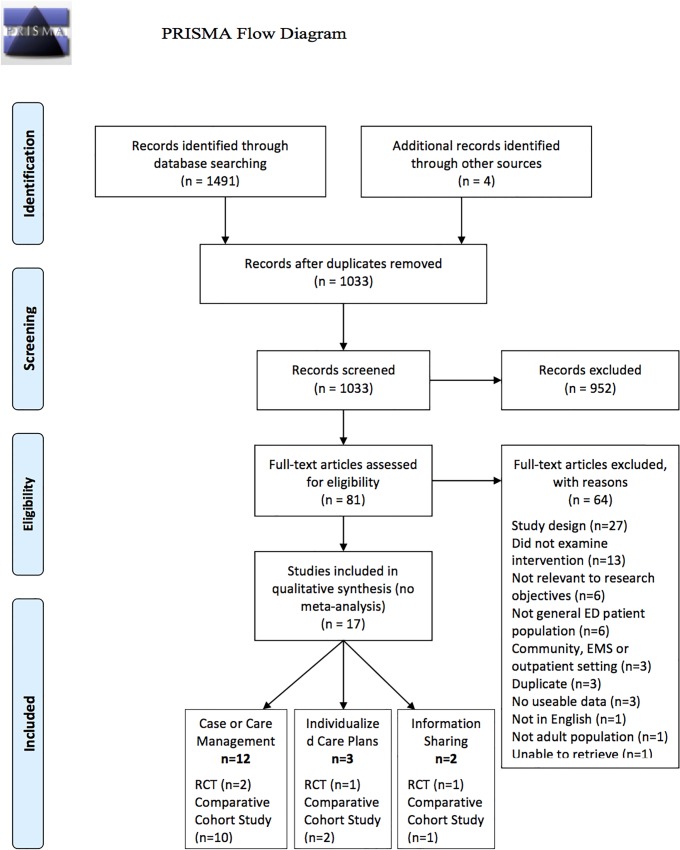
PRISMA Flow Diagram. A total of 1491 abstracts were identified from the electronic database search. After removal of duplicate records, 1029 abstracts were reviewed and 952 were excluded. Hand-searching of the references lists of relevant systematic reviews identified 4 additional full-text articles. Eighty-one articles in all were assessed in full-text, of which 64 were excluded and 17 studies (4 randomized controlled trials; 13 comparative cohort studies), within 3 intervention categories (care or case management; individualized care plans; information sharing), were included for final qualitative synthesis. Heterogeneity in the reported outcomes prohibited pooling of data for meta-analysis.

One thousand and twenty-nine citations were identified using the search strategy outlined above. Of those, 952 were excluded based on abstract review. The remaining 77 studies proceeded to full-text review. Systematic reviews were also hand-searched and 4 additional relevant articles were identified. Sixty-four were then excluded following full-text review and 17 articles were included in the final analysis (Kappa = 0.905, 95% CI 0.775–1.00) (Fig 1).

The characteristics of included studies are summarized in Table 1. Included articles were published between 1997 and 2014. The majority of studies were in the United States (n = 8);[18–20,23,28–32] three were conducted in Australia[21,33] and New Zealand;[34] two were conducted in Sweden;[35,36] one was conducted in Canada;[22] one was conducted in Scotland;[24] and one was conducted in Taiwan.[37] Four of the studies were RCTs[18,28,35,36] and the remaining 13 were designed as non-randomized comparative cohort studies.[19–24,29–34,37] The study follow-up periods ranged from 6 to 12 months post-intervention and the primary outcome of interest was the change in mean ED visits either between groups or before and after the intervention. Sixteen of the included studies were conducted in a single acute care facility; often a public and/or academic hospital in either an urban and rural setting.[18–24,29–37] The patient population in the remaining study[28] was drawn from 7 different acute care hospitals. Sample sizes of the RCTs varied from 25 to 1799 patients and from 14 to 258 patients for comparative cohort studies.

**Table 1:** Characteristics of Included Studies.

Author (Year) Country	Journal	Health Care Setting	Study Population	Type of Control	Number of Participants	Definition of Frequent User	Type of Intervention	Health Professionals involved in Intervention	Quality Assessment/ Score
**Randomized Controlled Trials**									
**Hansagi[35] (2008) Sweden**	Eur J Emerg Med	One large urban hospital ED	Patients with ≥ 3 visits in the last 12 months	Randomly assigned controls	Control: **965** Exposed: **834**	Patients with 3 or more ED visits in the last 12 months	**Information Sharing** given to physicians	ED Physicians, primary care providers	Medium Risk of Bias^§^
**Reinius[36] (2013) Sweden**	Eur J Emerg Med	One large urban hospital ED	Patients with ≥ 3 ED visits in the previous 6 months	Randomly assigned controls	Control: **57** Exposed: **211**	Three or more visits in 3 months	**Case management** using personalized programs and telephone contact	Nurses	Medium Risk of Bias^§^
**Shumway[18] (2008) United States**	Am J Emerg Med	One urban public hospital ED	Patients seen in ED ≥ 5 times in past 12 months and had psychosocial problems	Randomly assigned controls	Control: **85** Exposed: **167**	Patients with 5–11 ED visits in 12 months	**Case management** including group support, help with psychosocial problems, access to medical care providers	Psychiatric social workers	Medium Risk of Bias^§^
**Spillane[28] (1997) United States**	Acad Emerg Med	Seven hospital EDs	Adult patients with ≥ 10 ED visits	Randomly assigned controls	Control: **25** Exposed: **27**	Ten or more ED visits in 12 months	**Individualized care plan** including psychosocial evaluation, care coordination	ED physician, social worker, psychiatrist	Medium Risk of Bias^§^
**Comparative Cohort Studies**									
**Chiang[37] (2014) Taiwan**	Hong Kong J Emerg Med	One large urban hospital ED	Patients seen in the ED ≥ 3 times within 72 hours cases were divided into the pain management or chronic disease group according to their chief complaint.	Patients were used as their own controls (historical control)	Control: **14** Exposed: **14**	Three or more ED visits within a 72-hour period in a month	**Case management** using dynamic internet-mediated team-based support led by emergency physicians	ED physicians, primary care physicians, psychiatrists, social workers, and pharmacologists	14/26^†^
**Crane[29] (2012) United States**	The Journal of the American Board of Family Medicine	One not-for-profit hospital ED	Patients with low income who visited the ED ≥ 6 times in the past 12 months	Total sample population used as control group	Control: **36** Exposed: **36**	Six or more visits to ED in 12 months	**Case management** including telehealth line, drop-in group meetings, and life skills training	Family Physician, nurse care manger, behavioural health providers	17/26^†^
**Dehaven[30] (2012) United States**	J Public Health Med	One large urban hospital ED	Patients with low income who visited the ED ≥ 2 times in the past 12 months	All patients eligible for study who were not in intervention group	Control: **25** Exposed: **27**	Ten or more visits to ED in 12 months	**Individualized care plan** including access to a primary care provider	Primary care providers, hospital-based coordinator, in-person or telephone access to community health worker	20/26^†^
**Grimmer-Somers [33] (2010) Australia**	Aust N Z J Public Health	One urban hospital ED	Patients who consistently used public hospital EDs	Patients were used as their own controls (historical control)	Control: **37** *Exposed: **37** *	No definition given	**Individualized care plan** including health assessment, social support, and education	Social workers, nurses	15/26^†^
**Lee[19] (2006) United States**	The Health Care Manager	One ED in a teaching hospital	Patients with ≥ 3 ED visits per month	Patients were used as their own controls (historical control)	Control: **50** * Exposed: 50*	More than 3 visits to ED in one month	**Case management** including access to primary care physicians, and life skills assistance	Social workers, case managers, primary care physicians	19/26^†^
**Murphy[32] (2014) United States**	J Emerg Med	One regional hospital ED	Patients with ≥ 3 ED visits in past 12 months	Patients were used as their own controls (historical control)	Control: **65** * Exposed: **65** *	Three to eleven ED visits in 12 months pre-enrollment	**Case management** including multidisciplinary ED-care-coordination, individualized ED-care guidelines, and information system	Physicians, nurses, mental-health and substance-abuse professionals, ED nurse managers, a pharmacist, a social worker, a chaplain	19/26^†^
**Okin[20] (2000) United States**	Am J Emerg Med	One urban hospital ED	Convenience sample of frequent ED users (≥ 5 visits in 12 months)	Patients were used as their own controls (historical control)	Control: **53** * Exposed: **53** *	Five or more visits in 12 months	**Case Management** including service coordination, group support, housing arrangement, referrals, home visits	Psychiatric social worker, case manager, primary care physician	18/26^†^
**Peddie[34] (2011) New Zealand**	N Z Med J	One urban hospital ED	Patients with ≥ 10 ED visits in 12 months	Control group from Kennedy et al.[40]	Control: **77** Exposed: **87**	Ten or more visits to ED in 12 months	**Case Management** including medical evaluation, access to primary care physicians	Nurse, general practitioners, ED staff, psychiatric services, social workers, medical specialists	15/26^†^
**Phillips[21] (2006) Australia**	Med J Aust	One urban hospital ED	Patients with ≥ 6 ED visits in 12 months	Patients were used as their own controls (historical control)	Control: **60** * Exposed: **60** *	Six or more visits to ED in 12 months	**Case Management** including psychosocial evaluation, and access to health care practitioners	Nurses, allied health professionals, psychiatry, primary care providers	19/26^†^
**Pope[22] (2000) Canada**	CMAJ	One downtown hospital ED	Patient with frequent ED service use, in addition to violent behaviour and/or, drug-seeking behaviour and/or having a chronic medical condition	Patients were used as their own controls (historical control)	Control: **24** * Exposed: **24** *	No definition given	**Case management** including referrals to address social and medical needs	Social workers, ED medical director, director of continuous quality improvement, patient care manager, psychiatric nurse, clinical nurse specialist, family physicians, community care providers	18/26^†^
**Shah[23] (2011) United States**	Med Care	One hospital ED	Low income, uninsured adults who are frequent ED users (≥ 4 ED visits in 12 months)	Controls from the same patient population	Control: **160** Exposed: **98**	**≥ 4** ED visits in 12 months	**Care management** including access to primary care, insurance coverage, access to support services, and care coordination	Case managers, Primary care providers	21/26^†^
**Skinner[24] (2009) Scotland**	Emerg Med J	One urban hospital ED	Patients with high ED use	Controls from the same patient population	Control: **21** Exposed: **36**	Ten or more visits in 6 months	**Case management** including evaluation, referrals, and care plan	Clinical nurse specialist, psychiatrist, ED specialist registrar, ED consultant, social workers, housing officers	16/26^†^
**Stokes-Buzzelli[31] (2010) United States**	West J Emerg Med	One urban hospital ED	Patients with high ED use	Patients were used as their own controls (historical control)	Control: **36** * Exposed: **36** *	No definition given	**Information Sharing** including treatment plan, support with social and medical issues, access to health care providers through electronic medical records	ED attending physician, Medical social worker, Mental health social worker, psychologist, medical resident, clinical nurse specialist, student volunteer	20/26^†^

*Same participants in the control and exposed groups (historical controls);

^**§**^Risk of bias assessed using the Cochrane Risk of Bias Tool;

^†^Quality assessed using the Downs and Black Checklist

### Quality of Included Studies

Each of the included RCTs had areas where the risk of bias was high, low, and unclear (Table 1 and S2 Table). Risk of bias was consistently high across all four studies in the areas of allocation concealment, and blinding. Since the interventions involved discussion with the participants, blinding and allocation concealment were not possible. Furthermore due to the nature of the examined interventions, treatment allocation was not concealed, nor could the participants, personnel, or outcome assessors be blinded.

The comparative cohort studies included were of low to moderate quality wherein quality assessment scores ranged from 14 to 20 points (Table 1 and S3 Table). Although typically scored out of 28, a modified Downs and Black Checklist[27] was used since some of the questions did not apply to the comparative cohort design (i.e. randomization); thereby reducing the denominator to 26. Studies received poor scores for reporting of adverse events and blinding.

### Defining Frequent Users and Control Groups

In 16 of the included studies, frequent users were defined in the context of ED utilization (i.e. frequent ED users) with a threshold number of 3 to 10 ED visits within the 12 months prior to the study period. In the remaining comparative cohort study, frequent ED use was defined as 3 or more ED visits within a 72 hour period in the month prior to the study.[37] The control groups in the 4 RCTs were comprised of randomly assigned subjects not exposed to the intervention of interest. For the majority of the comparative cohort studies (n = 8) a pre- and post-intervention design was used, where the population exposed to the intervention served as their own historical control groups.[19–22,31–33,37] The control groups in the remaining studies (i.e. not exposed to the intervention) were either sampled from the same population as the intervention groups[23,24,30]—in one case utilizing the total sample population as the control group[29]—or sampled from the control group of previous work[34].

### Types of interventions

Three types of interventions aimed at reducing the number of ED visits by frequent users were identified from the literature review: case management (also referred to as care management),[18–24,29,32,34,36,37] individualized care plans,[28,30,33] and information sharing.[31,35] Brief accounts of the specific interventions or combination of interventions as well as the composition of resources related to each intervention, as described by the included studies, are provided in Table 1.

### Case Management

Broadly defined, case or care management (CM) is considered a comprehensive, interdisciplinary approach taken to assess, plan, personalize, and guide an individual’s health services to promote improved patient and health system outcomes.[19] A single point of contact (e.g. an individual described as either a case manager, care manager, or ED consultant) is assigned to a frequent ED user and is tasked with brokering access and guiding the patient through their customized care process, which may extend beyond the normal continuum of the ED and in-patient care, into the community.[5]

Among the 12 studies (2 RCTs; 10 comparative cohort studies) that examined CM as an intervention for frequent ED users (see Table 1), interdisciplinary teams consisting of case managers, primary care physicians, nurses, social workers, other allied health, and ED physicians and staff were identified to implement the proposed CM intervention.[18–24,29,32,34,36,37] The specific approaches employed for each CM intervention varied across studies and included a variety of health services such as crisis intervention, supportive therapy, linkage to primary care and other medical providers, referral to substance abusive services, regular CM meetings and needs assessments, and assistance with obtaining stable income and housing.

The mean or median number of ED visits before and after the CM intervention for the included studies are summarized in Table 2. Compared to the control groups, one RCT reported no change in the mean number of ED visits following CM,[18] whereas the second RCT reported a minor decrease in median ED visits among those in the intervention group.[36] Of the 10 comparative cohort studies evaluating a CM intervention, nine studies reported outcomes related to the change in ED visits: eight studies observed a decrease in the mean (between -0.66 and -37 ED visits) [19,37] or median number of ED visits (between -2.28 and -20 ED visits) compared to the controls[29] or before CM;[20,22–24,32] and 1 study reported an increase of 2.79 median ED visits post-intervention.[21]

**Table 2:** Reported Outcomes of Frequent User Interventions Among Included Studies.

Authors	Type of Intervention	Control Group			Intervention Group		
		Number of ED Visits Before (mean per year)	Number of ED Visits After (mean per year)	Change in Mean Number of ED Visits	Number of ED Visits Before Intervention (mean per year)	Number of ED Visits After Intervention (mean per year)	Change in Mean Number of ED Visits
**Randomized Controlled Trials**							
Hansagi[35]	Information Sharing	6	3.9	-2.1	6.2	4	-2.2^§^
Reinius[36]	Case management	5*	6.4*	+1.4*	5*	4.9*	-0.1*
Shumway[18]	Case management	5.2	2.9	-2.3	3.6	1.4	-2.2
Spillane[28]	Individual care plan	13*	6*	-7*	14*	7*	-7*
**Comparative Cohort Studies**							
Chiang[37]	Case management	-	-	-	63^§^	26^§^	-37^§^
Crane[29]	Case management	6.96*	5.04*	-1.92*	6.96*	2.76*	-4.2*
Dehaven[30]	Individual care plan	-	1.44	-	-	0.93	-
Grimmer-Somers[33]	Individual care plan	-	-	-	-	-	-
Lee[19]	Case management	-	-	-	8.92	8.26	-0.66
Murphy[32]	Case management	-	-	-	7*	2*	-5*
Okin[20]	Case Management	-	-	-	15*	9*	-6*
Peddie[34]	Case Management	-	14.6	-	-	17.1	-
Phillips[21]	Case Management	-	-	-	10.16	12.95	+2.79
Pope[22]	Case management	-	-	-	26.5*	6.5*	-20*
Shah[23]	Care management	-	-	-	6	1.7	-4.3*
Skinner[24]	Case management	-	-	-	12* ^§^	6* ^§^	-6* ^§^
Stokes-Buzzelli[31]	Information Sharing	-	-	-	67.4	50.5	-16.9

*Median reported;

^**§**^6 month time period

Of these 12 included studies, six (2 RCT and 4 comparative cohort studies) also assessed the change or difference in cost following the implementation of CM.[18,20,23,29,32,36] The results of these studies are summarized in Table 3. The two RCTs specifically assessed the costs of CM programs from a health system perspective. Shumway *et al*. reported increases in the cost of care for all participants in the 12-month follow-up period.[18] This increase in cost, however, was significantly less among those exposed to the CM intervention compared to those in the control group (CM: $3116 added costs per-patient vs. control: $6659 added costs per-patient; *p*<0.01).[18] The specific cost of the CM intervention was also reported as $606,711, or $3,633 per-patient.[18] The RCT conducted by Reinius *et al*. only reported the health system costs following a telephone-based CM intervention (i.e. direct cost of implementing the CM intervention was not reported).[36] The total costs per-patient were reported as €6,355 for the intervention group and €19,044 for the control group; this estimated 45% decrease in cost was found to be statistically significant (*p* = 0.004).[36]

**Table 3:** Health System Costs of Frequent ED Use and/or Interventions.

Author	Country	Intervention Type	Number of Included Participants	Cost of Intervention	Hospital Charges Before Intervention (per patient)	Hospital Charges After Intervention (per patient)	Change in Hospital Charges (per patient)	Change in Total Hospital Charges
**Randomized Controlled Trials**								
Shumway[18]	United States	Case management	Control group: **85** Exposed: **167**	$606,711 ($3,633 per patient)	Intervention Group: $11,805 Control Group: $10,020	Intervention Group: $14,921 Control Group: $16,679	Intervention Group: $3,116 Control Group: $6,659	-
Reinius[36]	Sweden	Case management	Control group: **57** Exposed: **211**	-	-	Intervention Group: €6,355 Control Group: €19,044	-	-
**Comparative Cohort Studies**								
Crane[29]	United States	Case management	Control group: **36** Exposed: **36**	$66,000 ($1,833 per patient)	$1,167	$230	-$937	-
Dehaven[30]	United States	Individualized care plan	Control group: **25** Exposed: **27**	-	$1188	$446	-$742	-
Grimmer-Somers[33]	Australia	Individualized care plan	Control group: **37** Exposed: **37**	$63,434 ($1,714 per patient)^†^	-	-	-	-
Murphy[32]	United States	Case management	Control group: **65** Exposed: **65**	$265,680	$2,328*	$1,043*	-$1,285	-$142,809
Okin[20]	United States	Case management	Control group: **53** Exposed: **53**	$296,738 ($5,599 per patient)	$12,454*	$4,981*	-$7,473	-$429,464
Shah[23]	United States	Care management	Control group: **160** Exposed: **98**	-	$2,545^§^	$1,874^§^	-$671^§^	
Stokes-Buzzelli[31]	United States	Information sharing	Control group: **36** Exposed: **36**	-	-	-	-$15,513^§^	-

*Median reported;

^**§**^ED charges only;

^†^In Australian Dollars.

Four comparative cohort studies also evaluated the cost of CM from a health system perspective (Table 3).[20,23,29,32] Broadly, all four studies reported reduced hospital costs (i.e. ED and in-patient charges) per patient in the 12-months following a CM intervention. The Shah *et al*. study reported a modest decrease of $671 in hospital charges per patient.[23] Whereas the greatest reduction in median per-patient hospital costs was $7,473, reported by the Okin *et al*. study; this resulted in a cost savings of $429,464 in the hospital charges for the entire intervention group (n = 53).[20] This study also reported a net cost savings (i.e. the cost of the intervention was subtracted from the savings due to the intervention) of $132,726.[20] Lastly, costs specifically related to the implementation of the CM programmes were reported by 4 studies (1 RCT; 3 comparative cohort studies) and ranged from $66,000 (or $1,833 per patient) to $606,711 ($3,633 per patient) for CM.[18,20,29,32]

### Individualized Care Plan

One RCT and two comparative cohort studies examined the impact of individualized care plans on ED visitation rate amongst frequent ED users.[28,30,33] Similarly to CM, individualized care plans employ interdisciplinary strategies, including cross-departmental care plan meetings[28] and coordinated access to primary care resources.[30,33] However in contrast to the CM approach, individualized care plans were described as less comprehensive in their design, limited in the number of health services and, importantly, implemented without a designated case manager or equivalent.

The one RCT which evaluated implementation of individualized care plans found no difference in the median number of ED visits between intervention and control groups 12 months post-intervention (care plan group: -7 ED visits; control groups: -7 ED visits).[28] The two comparative cohort studies did not report outcome data related to the frequency of ED visitations following care plan implementation (Table 2).[30,33]

Of these 3 included studies, the two comparative cohort studies provided cost-related outcomes from a health system perspective (Table 3).[30,33] The first study reported a reduction in hospital charges of $742 per patient following the implementation of individualized care plans.[30] The second study did not provide the change in per patient hospital costs, yet reported the implementation costs of the individualized care plan program which was $63,434 Australia Dollars (AUD) or $1,714 AUD per patient.[33]

### Information sharing

The term ‘information sharing’ was used to describe approaches related to the sharing of patient information (clinical and/or demographic information) amongst health care providers. Two studies (1 RCT and 1 comparative cohort study) evaluated the implementation of different information sharing strategies to frequent ED user populations.[31,35]

The RCT implemented an information sharing strategy wherein ED and primary care physicians shared access to printed case notes, through an electronic database system, of each frequent user patient.[35] Six months post-intervention, this form of information sharing did not result in a significant difference in the mean number of ED visits between treatment groups (information sharing group: -2.2; control group: -2.1) (Table 2). No cost-related outcomes were reported for this study.[35]

The comparative cohort study by Stokes-Buzzelli *et al*. utilized an electronic medical records (EMR) system to share individualized care plans between relevant ED physicians and allied health practitioners (e.g. medical and mental health social workers, psychologist, and nurse specialists).[31] This intervention reportedly led to increased consistency in identifying and managing frequent ED users and, ultimately, in a significant decrease in mean ED visits 12-months following the intervention (from 67.4 to 50.5 mean ED visits; *p* = 0.046) (Table 2). This study also reported a substantial reduction of $15,513 per patient in ED costs in the 12-months follow-up period.[31] The cost of the intervention, however, was not reported by this study (Table 3).

## Discussion

This systematic review identified 17 low to moderate quality studies evaluating the impact of interventions targeted to reduce frequent ED use. Three categories of interventions were identified: case or care management, individualized care plans and information sharing systems. Case or care management, as well as individualized care plan interventions, were consistently reported to result in reduced hospital charges and no studies reported of increases in hospital charges post-intervention. However, reductions in ED visit number were also observed among control groups in 3 of the 4 RCTs.[18,28,35] Significant costs and resources were also reportedly required for implementation of the examined intervention and the present evidence indicates that none are likely to yield substantial, overall cost savings for the healthcare system.

Of the three interventions reported in the literature, the interdisciplinary approach of CM was the most widely evaluated (2 RCTs; 10 comparative cohort studies). Despite the breadth of this evidence base, use of CM resulted in variable reductions in frequent ED use. The two RCTs that evaluated CM interventions demonstrated minor changes in mean and median number of ED visits between the intervention and control groups (ranging from +0.1 to -1.5 ED visits).[18,36] Whereas among comparative cohort studies reporting the primary outcome of interest, seven studies reported reductions of greater than 4 annual ED visits compared to the control settings (e.g. mean: -37 visits; median: -4.2 to -20 ED visits).[20,22–24,29,32,37] For example, the comparative cohort study conducted by Pope *et al*. reported the greatest reduction in median annual ED visits (from 26.5 to 6.5 ED visits) following a CM program for a small sample (n = 24) of frequent users.[22] The authors described the intervention as a ‘difficult’ CM targeted at the needs of frequent ED users from an impoverished area of an urban centre, with chronic and complex medical conditions, drug-seeking tendencies, and exhibiting violent and/or abusive behaviours.[22] Given the non-randomized nature of these studies and the small, selective study populations, there may be other uncontrolled confounding or modifying factors influencing the observed reduction in ED utilization. Therefore the extent of effectiveness of CM demonstrated by these comparative cohort studies must be cautiously interpreted.

The limited evidence base for the remaining two frequent user intervention categories was somewhat inconclusive. Interestingly, among studies reporting the primary outcome of interest, there was a similar relationship in demonstrable effectiveness and study design. Studies conducted within a higher quality RCT setting reported no change in median or mean ED visits following individualized care plans[28] or information sharing,[35] respectively. Whereas the one comparative cohort study reported a marked decrease in the mean number of ED visits (-16.9).[31] In this latter case, the intervention was again only applied to a small, selective sample of frequent users in a non-randomized setting. Thus, similar to those comparative cohort studies evaluating CM, with concern over the internal validity the results of the Stokes-Buzzelli *et al*. study must be interpreted with caution.[31]

While less commonly reported, there was also evidence to support use of multi-modal frequent ED user interventions. The Murphy and Neven study, for example, coordinated their multi-disciplinary case management programme using a regional hospital information system that allowed participating EDs to view the care guidelines developed for each frequent user participant.[32] Such automated access to patient records improved the efficiency of information exchange among physicians not just in the ED, but across the continuum of care.[32] Similarly, Stokes-Buzzelli *et al*. developed care plans that were uploaded onto an information sharing system (i.e. EMR system) accessible to necessary healthcare providers.[31] This combinatorial approach led to more efficient and consistent identification and management of frequent ED users.[31] Interestingly, the care plan component of Stokes-Buzzelli *et al*. appeared to closely resemble the CM approaches described among included studies (e.g. creation of individualized care programs and formation of a committee of ED clinicians), but was led by the care committee rather than an individual case manager.[31]

Based on the limited cost-related outcomes reported, implementation of the examined interventions was expensive. Case management, in particular, can incur high health system costs as elements of crisis intervention, supportive therapy, primary and secondary care, substance abuse services, community outreach, housing support,[5] and a multidisciplinary CM team (i.e. case manager, ED and primary care physicians, nurses, social workers, other allied health and ED staff) are often included. Among the two cost-effectiveness studies conducted, the cost of implementing a CM programme was approximately $606,711($3,633 per patient) per year in a RCT and $265,680 within a comparative cohort study setting.[18,32] It is important to note, however, that limited reporting of costing data (e.g. incurred from the intervention, resource utilization) alongside efficacy or effectiveness data for all three intervention types precluded conclusive determination of the most cost-effective strategy. There was also only limited evidence to infer the potential cost-savings from the examined interventions. The Shumway *et al*. RCT, for example, reported that the CM intervention resulted in lower hospital costs during the 12-month follow-up period when compared to the control setting (CM: $3116 added costs vs. control: $6659 added costs).[18] Stokes-Buzzelli *et al*. determined that following their information sharing intervention (which incurred costs due to use of EMR and individualized care plans), there was an overall reduction of $15,513 in hospital charges for the entire intervention group (n = 36).[31] In light of the gaps and variability of evidence reported, further budget impact analyses and cost-effectiveness studies—adopting both health system and societal perspectives—are required to advise healthcare decision-makers of which frequent ED user intervention may prove most cost-effective.

The findings from this systematic review are consistent with those previously reported in two systematic reviews of in-hospital frequent ED user interventions.[5,6] The first of these reviews included literature published only up to 2010 and summarized the evidence concerning interventions for general, adult frequent ED users from 11 studies.[5] The second, more recent, review exclusively evaluated the effectiveness of CM strategies from 12 studies of both general and subgroups of frequent ED user populations.[6] Given the similarities to the search strategies employed in the previous reviews, there was considerable overlap in included studies as well as conclusions drawn with regards to efficacy or effectiveness and cost savings of interventions. Both reviews also reported of considerable heterogeneity in patient populations, intervention types, and outcomes amongst studies evaluated, similarly prohibiting pooling of results for meta-analysis. The present review is an updated account of the evidence base and provides a more comprehensive review of the intervention types. This is particularly relevant in light of the rapidly evolving literature concerning novel and more defined high use and high cost patient populations.[9,10,38,39] The present work also provides a more extensive account of cost analyses amongst included studies (reported in 9 of the 17 included studies).

There were several limitations to this review and the included literature that warrant discussion. In an attempt to focus on the literature concerning the general adult frequent user populations, studies that did not examine a general frequent ED user population (e.g. pediatric or senior populations, homeless individuals, individuals with a lower socioeconomic status, substance abuse, individuals with asthma, headache/migraine, sickle cell, diabetes, and cardiovascular disease) or focused on a specialized, community or out-patient care setting, were excluded. Studies with either a RCT or non-randomized prospective, comparative cohort observational design were exclusively selected for synthesis in this report, as these were deemed to be of the highest in study design quality. Also, since the majority of included studies were conducted within the United States, the generalizability of these study findings to other international healthcare contexts remains unclear. The quality and availability of data from the included studies also proved to be problematic for final analyses. Each of the included RCTs had areas where the risk of bias was high, low, and unclear (S2 Table). Risk of bias was consistently high across all four studies in the areas of allocation concealment, and blinding. However, due to the nature of these interventions, it was not possible to blind allocation, participants, personnel or outcome assessment and, therefore, all four studies received a high risk of bias assessment in these areas. The majority of the included comparative cohort studies—which were of low to moderate quality—also employed a pre- and post- intervention design wherein individuals acted as their own controls (historical control cohort). Therefore, in those instances, randomization was not possible. Collectively, the moderate to low quality of these studies and the heterogeneity in the composition of the study populations, the study or specific healthcare settings, the types of interventions and/or comparators, as well as inconsistent reporting of specific outcome measures, precluded meta-analyses. The clinical efficacy/effectiveness of interventions was thus arbitrarily gleaned from studies demonstrating reductions in ED visits post-intervention.

## Conclusions

Based on the literature evaluated in the present systematic review, three types of interventions have been evaluated: case management, individualized care plans and information sharing. The impact of the three types of frequent ED user interventions was variable, but modest at best. Case management had the most rigorous evidence base, yielded moderate cost savings, but with variable reductions in frequent ED use. The most clinically beneficial and cost-effective intervention to deter frequent ED use remains unclear given the overall variability in reported outcome and cost data. Considering the significant costs and resources required for implementation, the present evidence suggests that none of the examined interventions are likely to yield substantial, overall cost savings for the healthcare system. Findings from the present review further indicate that prior to implementing any given intervention, thorough identification of prevalent risk factors of frequent ED use, among ED populations, must first be conducted to determine inefficiencies or gaps in the delivery of health services and the resultant appropriateness of interventions. Such personalizing and tailoring of interventions and models of care, rather than standardization of care, may prove to be most effective at reducing high ED utilization.

## Supporting Information

S1 PRISMA Checklist Preferred Reporting Items for Systematic Reviews and Meta-Analyses (PRISMA) Checklist. (DOC)

S1 Table Sample Search Strategy (1950 to January 2015). (DOCX)

S2 Table Cochrane Risk of Bias Quality Assessment. (DOCX)

S3 Table Downs and Black Quality Assessment of Comparative Cohort Studies (DOCX)
