# Supplementary material for: Reducing Frequent Visits to the Emergency Department: A Systematic Review of Interventions
Source: PLoS One. 2015 Apr 13;10(4):e0123660. doi: 10.1371/journal.pone.0123660 (PMC4395429; doi:10.1371/journal.pone.0123660)
Supplement: S1 Table — (DOCX) [file pone.0123660.s002.docx]

**S1 Table: Sample Search Strategy (1950 to January 2015)**

| **EMBASE (OVID)** |
| --- |
| 1. exp hospital utilization/ or emergency health service/ |
| 2. (emergency department or health care or health care or health service* or clinic*).ti. |
| 3. 1 or 2 |
| 4. (overutiliz* or frequent user* or super-user* or over-user*).tw. |
| 5. 3 and 4 |
| 6. health service misuse.mp. |
| 7. 4 and 6 |
| 8. (overutiliz* adj10 (healthcare or health care or health service* or emergency or clinic or ED)).tw. |
| 9. (((frequent adj3 user*) or frequent ED or frequent emergency or super-user* or over-user*) adj10 (emergency or ED or healthcare or health care or clinic* or health service)).tw. |
| 10. 5 or 7 or 8 or 9 |
| 11. limit 10 to english language |
| 12. limit 11 to (editorial or letter) |
| 13. 11 not 12 |
| 14. limit 13 to "review" |
| 15. (systematic or meta-analyses or metaanalysis).ti. |
| 16. 14 and 15 |
| 17. 13 not 14 |
| 18. 16 or 17 |
