# Supplementary material for: Reducing Frequent Visits to the Emergency Department: A Systematic Review of Interventions
Source: PLoS One. 2015 Apr 13;10(4):e0123660. doi: 10.1371/journal.pone.0123660 (PMC4395429; doi:10.1371/journal.pone.0123660)
Supplement: S2 Table — (DOCX) [file pone.0123660.s003.docx]

**S2 Table:** Cochrane Risk of Bias Quality Assessment

| **Author** | **Random Sequence Generation** | **Allocation Concealment** | **Blinding of Participants and Personnel** | **Blinding of Outcome Assessment** | **Incomplete Outcome Data** | **Selective Reporting** | **Other Bias** |
| --- | --- | --- | --- | --- | --- | --- | --- |
| **Hansagi^33^** | Low Risk | High Risk | High Risk | High Risk | Low Risk | Low Risk | Unclear Risk |
| **Reinius^34^** | Low Risk | High Risk | High Risk | High Risk | Low Risk | Low Risk | Unclear Risk |
| **Shumway^18^** | Low Risk | High Risk | High Risk | High Risk | Low Risk | Low Risk | Unclear Risk |
| **Spillane^27^** | Low Risk | High Risk | High Risk | High Risk | Low Risk | Low Risk | Unclear Risk |
