# Supplementary material for: Reducing Frequent Visits to the Emergency Department: A Systematic Review of Interventions
Source: PLoS One. 2015 Apr 13;10(4):e0123660. doi: 10.1371/journal.pone.0123660 (PMC4395429; doi:10.1371/journal.pone.0123660)
Supplement: S3 Table — (DOCX) [file pone.0123660.s004.docx]

**S3 Table:** Downs and Black Quality Assessment of Comparative Cohort Studies

|  | | **DeHaven** [30] | **Stokes-Buzzelli** [31] | **Crane**[21] | | **Peddie** [34] | **Shah**[23] | **Grimmer-Somers** [33] | **Phillips** [21] | **Lee**[19] | | **Okin**[20] | **Pope**[22] | **Skinner** [24] | **Chiang** [37] | **Murphy** [32] |
| --- | --- | --- | --- | --- | --- | --- | --- | --- | --- | --- | --- | --- | --- | --- | --- | --- |
| **REPORTING** | | | | |  | | | | | |  | | | | | |
| **Q1** | **Hypothesis/aim/objective clearly described** | 1 | 1 | 1 | | 1 | 1 | 1 | 1 | 1 | | 1 | 1 | 1 | 1 | 1 |
| **Q2** | **Main outcomes in Introduction or Methods** | 1 | 1 | 1 | | 1 | 1 | 1 | 1 | 1 | | 1 | 1 | 1 | 1 | 1 |
| **Q3** | **Patient characteristics clearly described** | 1 | 1 | 1 | | 1 | 1 | 1 | 1 | 1 | | 1 | 1 | 1 | 1 | 1 |
| **Q4** | **Interventions of interest clearly described** | 1 | 1 | 1 | | 1 | 1 | 1 | 1 | 1 | | 1 | 1 | 1 | 1 | 1 |
| **Q5** | **Principal confounders clearly described** | 1 | 0 | 0 | | 0 | 2 | 0 | 1 | 2 | | 0 | 0 | 0 | 0 | 0 |
| **Q6** | **Main findings clearly described** | 0 | 1 | 1 | | 1 | 1 | 0 | 1 | 1 | | 1 | 1 | 1 | 1 | 1 |
| **Q7** | **Estimates of random variability provided for main outcomes** | 1 | 1 | 0 | | 1 | 1 | 0 | 1 | 1 | | 1 | 1 | 1 | 0 | 1 |
| **Q8** | **All adverse events of intervention reported** | 0 | 0 | 0 | | 0 | 0 | 0 | 0 | 0 | | 0 | 0 | 0 | 0 | 0 |
| **Q9** | **Characteristics of patients lost to follow-up described** | 1 | 1 | 1 | | 1 | 1 | 0 | 1 | 0 | | 1 | 1 | 1 | 1 | 1 |
| **Q10** | **Probability values reported for main outcomes** | 1 | 1 | 1 | | 0 | 1 | 1 | 1 | 1 | | 1 | 1 | UTD | 0 | 0 |
| **EXTERNAL VALIDITY** | | | | |  | | | | | |  | | | | | |
| **Q11** | **Subjects asked to participate were representative of source population** | 1 | 1 | 0 | | UTD | UTD | 0 | 0 | 0 | | 0 | 0 | 0 | UTD | 1 |
| **Q12** | **Subjects prepared to participate were representative of source population** | 1 | 1 | 0 | | UTD | UTD | 0 | 0 | 0 | | 0 | 0 | 0 | UTD | 1 |
| **Q13** | **Location and delivery of study treatment was representative of source population** | 1 | 1 | 1 | | 1 | 1 | 1 | 1 | 1 | | 1 | 1 | 1 | 1 | 1 |
| **INTERNAL VALIDITY – BIAS & CONFOUNDING** | | | | |  | | | | | |  | | | | | |
| **Q14** | **Study participants blinded to treatment** | 0 | 0 | 0 | | 0 | 0 | 0 | 0 | 0 | | 0 | 0 | 0 | 0 | 0 |
| **Q15** | **Blinded outcome assessment** | 0 | 0 | 0 | | 0 | 0 | 0 | 0 | 0 | | 0 | 0 | 0 | 0 | 0 |
| **Q16** | **Any data dredging clearly described** | 1 | 1 | 1 | | 1 | 1 | 1 | 1 | 1 | | 1 | 1 | 1 | 1 | 1 |
| **Q17** | **Analyses adjust for differing lengths of follow-up** | 1 | 1 | 1 | | 1 | 1 | 1 | 1 | 1 | | 1 | 1 | 1 | 1 | 1 |
| **Q18** | **Appropriate statistical tests performed** | 1 | 1 | 1 | | 1 | 1 | 1 | 1 | 1 | | 1 | 1 | 1 | 0 | 1 |
| **Q19** | **Compliance with interventions was reliable** | 1 | 1 | 1 | | 1 | 1 | 1 | 1 | 1 | | 1 | 1 | 1 | 1 | 1 |
| **Q20** | **Outcome measures were reliable and valid** | 1 | 1 | 1 | | 1 | 1 | 1 | 1 | 1 | | 1 | 1 | 1 | 1 | 1 |
| **Q21** | **All participants recruited from the same source population** | 1 | 1 | 1 | | 0 | 1 | 1 | 1 | 1 | | 1 | 1 | 1 | 1 | 1 |
| **Q22** | **All participants recruited over the same time period** | 1 | 1 | 1 | | 0 | 1 | 1 | 1 | 1 | | 1 | 1 | 1 | 1 | 1 |
| **Q23** | **Participants randomized to treatment(s)** | N/A | N/A | N/A | | N/A | N/A | N/A | N/A | N/A | | N/A | N/A | N/A | N/A | N/A |
| **Q24** | **Allocation of treatment concealed from investigators and participants** | N/A | N/A | N/A | | N/A | N/A | N/A | N/A | N/A | | N/A | N/A | N/A | N/A | N/A |
| **Q25** | **Adequate adjustment for confounding** | 0 | UTD | UTD | | UTD | 1 | 0 | UTD | 1 | | UTD | 0 | 0 | UTD | UTD |
| **Q26** | **Losses to follow-up taken into account** | 1 | 1 | 1 | | 1 | 1 | 1 | 1 | UTD | | 1 | 1 | UTD | 1 | 1 |
| **POWER** | | | | |  | | | | | |  | | | | | |
| **Q27** | **Sufficient power to detect treatment effect at significance level of 0.05** | 1 | 1 | 1 | | 1 | 1 | 1 | 1 | 1 | | 1 | 1 | 1 | 0 | 1 |
| **TOTAL** | | **20/26** | **20/26** | **17/26** | | **15/26** | **21/26** | **15/26** | **19/26** | **19/26** | | **18/26** | **18/26** | **16/26** | **14/26** | **19/26** |

UTD: Unable to determine; N/A: Not applicable.
